# Use of an Intracranial Drain as a Conduit for Treatment of an Intracranial Streptococcus intermedius Abscess

**DOI:** 10.7759/cureus.14613

**Published:** 2021-04-21

**Authors:** Shoeb B Lallani, Melanie Hyte, Emily Trieu, Carlos Reyes-Sacin, Ninh Doan

**Affiliations:** 1 Neurology, University of Alabama at Birmingham School of Medicine, Birmingham, USA; 2 Pharmacology, Baptist Medical Center South, Montgomery, USA; 3 Neurosurgery, Pleasant Grove High School, Elk Grove, USA; 4 Infectious Disease, Baptist Medical Center South, Montgomery, USA; 5 Department of Neurosurgery, Medical College of Wisconsin, Milwaukee, USA

**Keywords:** intracranial abscess, antibiotic administration, neurosurgery, microbiology, infectious disease

## Abstract

Brain abscesses are difficult to manage clinically and often result in a poor outcome. Although surgical and medical therapeutics have progressed, there are still challenges that make treating intracranial abscesses problematic. One of these treatment barriers is the poor penetration of intravenous antibiotics to the infection source through the blood-brain barrier. In this case report, we will discuss the use of a surgical drain as a conduit for direct antibiotic administration for a rare, recurrent *Streptococcus intermedius *infection. This technique allows us to bypass the blood-brain barrier while also reducing the systemic effects of antibiotics. When used in conjunction with craniotomy and resection, direct antibiotic administration via a surgical drain proved to be effective at treating our patient’s abscess and preventing recurrence.

## Introduction

Intracranial abscess is a devastating diagnosis with a high rate of morbidity and mortality that affects a wide range of populations. Even with the advent of new technologies and approaches to target these abscesses, there remains a 10% mortality rate within the general population [1,2]. One of the difficulties with current methods for treatment of intracranial abscesses is proper access to the lesion site. It can often be difficult to achieve adequate levels of antibiotics at the lesion via an intravenous introduction without causing systemic toxicity [3]. In this article, we explore a case study employing an intracranial drain as a conduit for direct drug administration in the treatment of a rare *Streptococcus intermedius* infection.

## Case presentation

**Figure 1:**
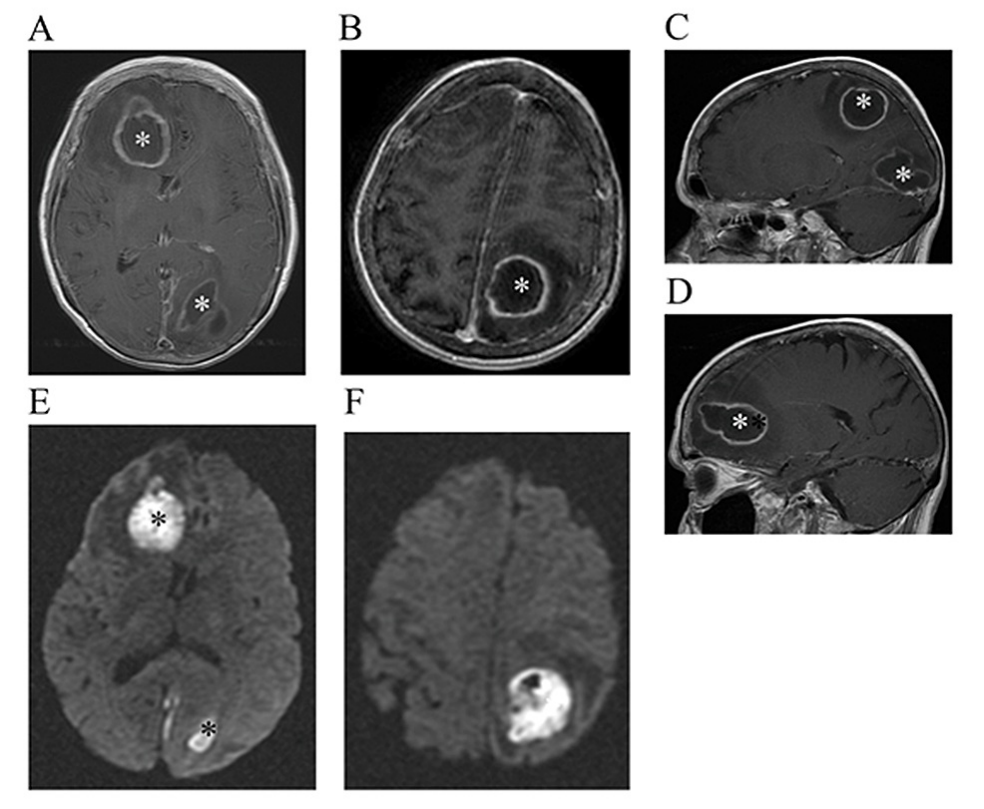
Initial MRI with contrast. Initial MRI of the brain with contrast (A-D) and DWI (E-F) showing the three large enhancing lesions in frontal, occipital, and parietal areas with DWI signals consistent with the presence of brain metastasis or abscesses (*). MRI: magnetic resonance imaging; DWI: diffusion-weighted imaging

A 70-year-old female presented to her primary care physician complaining of headaches. Her primary care physician gave her an initial diagnosis of sinusitis and prescribed her an intramuscular corticosteroid injection, but her headache continued to worsen. Over the next week, the patient stopped talking, eating, and developed right-sided weakness. Due to this progression of symptoms, magnetic resonance imaging (MRI) of the brain was ordered which revealed multiple cerebral ring-enhancing lesions, with the largest lesion measuring 4.6 cm in the right frontal lobe white matter. There was a 3.6 cm lesion in the left parietal lobe white matter and another 3.6 cm lesion in the left occipital lobe. All lesions demonstrated surrounding vasogenic edema, local mass effect, increased internal T2 signal, and restricted diffusion (Figure 1). The patient was taken to surgery for craniotomy and resection of the frontal and left parietal abscesses. Specimens for culture were taken intraoperatively. Final culture was positive for *S. intermedius*, sensitive to ceftriaxone (minimum inhibitory concentration of 0.12 μg/mL), clindamycin (0.06 μg/mL), levofloxacin (0.5 μg/mL), linezolid (1.0 μg/mL), and penicillin (0.03 μg/mL).

**Figure 2:**
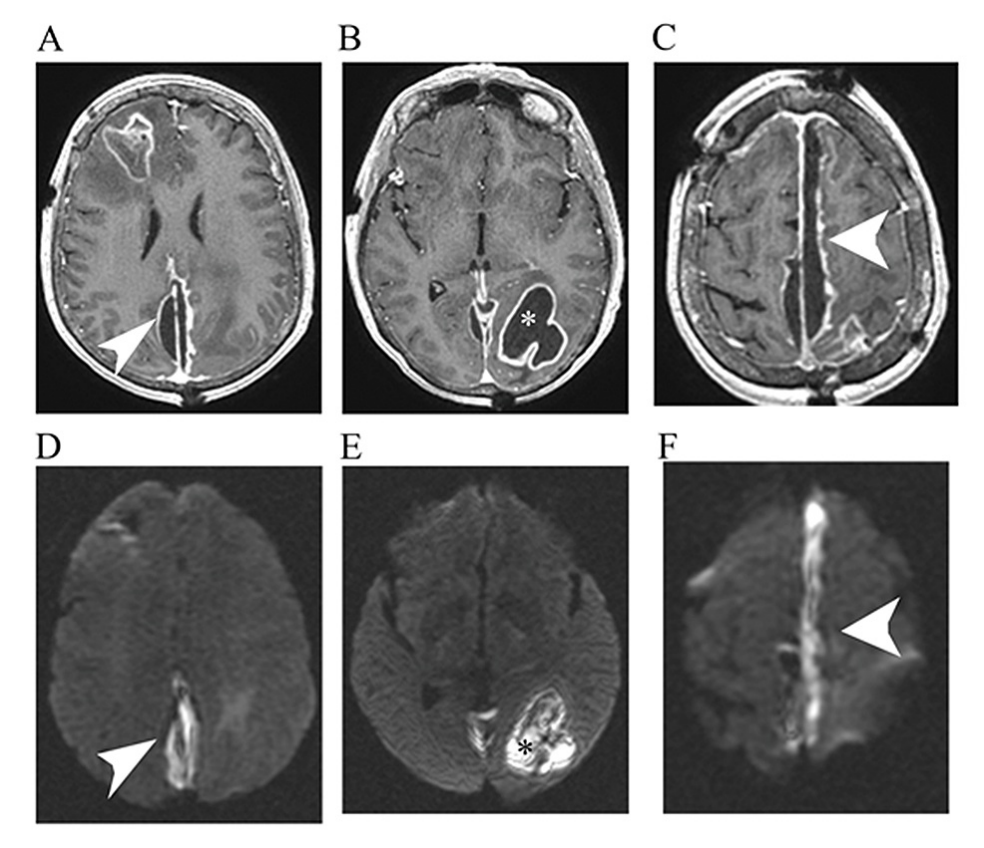
Repeat MRI with contrast T1. MRI of the brain axial with contrast T1 sequence (A-C) and DWI sequence (D-F) with new subdural empyema (arrows) and worsening of the occipital abscess are shown, suspected of communications between these lesions. MRI: magnetic resonance imaging; DWI: diffusion-weighted imaging

**Figure 3:**
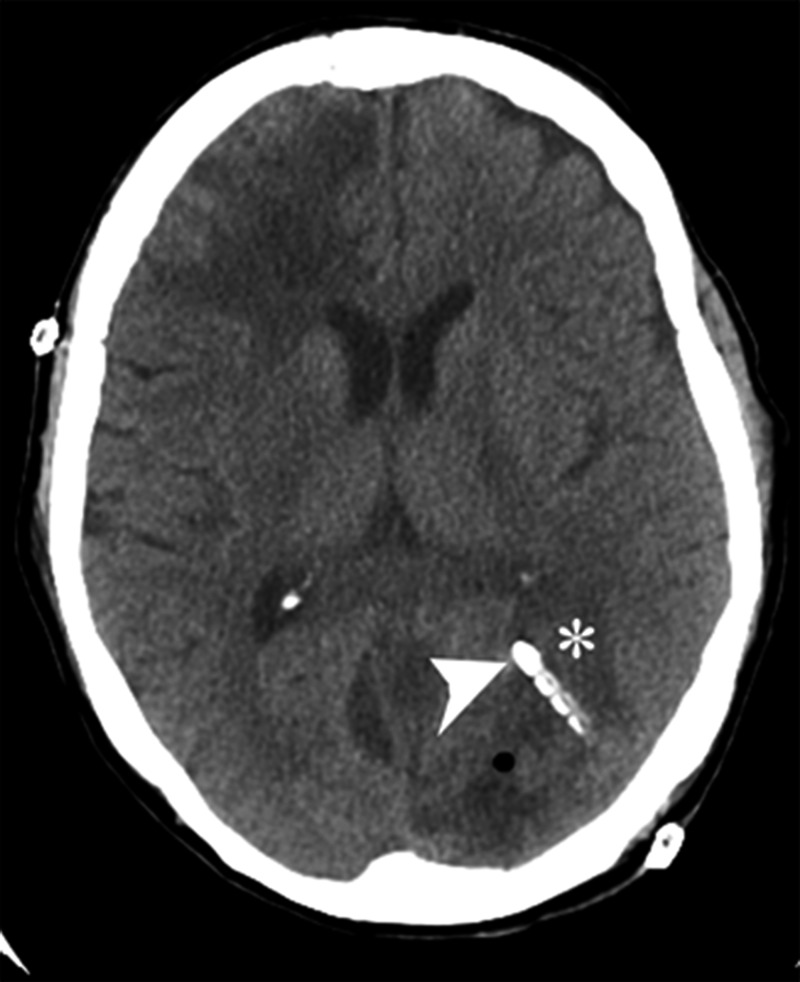
CT brain without contrast showing abscess and catheter. CT of the brain without contrast is shown with the abscess (*) penetrated by the catheter (arrow), through which vancomycin was injected for direct intra-abscess antibiotic treatment. CT: computed tomography

Her mentation improved after the surgery and she was discharged to an extended care facility where she was treated with 2 g of intravenous ceftriaxone every 12 hours for six weeks. While at the facility, she was noted to be febrile and demonstrated a decline in mentation by the house staff. She was transported back to the emergency department, where she was found to be lethargic and globally aphasic, with worsening right-sided weakness, a fever of 101.9°F, and an elevated white blood cell count of 29.6 × 10^3^. Computed tomography (CT) and MRI of the brain with and without contrast showed a new, large epidural abscess along the superior interhemispheric fissure between the frontal and parietal lobes, measuring 20 mm in thickness. There was also worsening of the multifocal cerebral abscess in the left parieto-occipital lobe (Figures 2, 3).

**Figure 4:**
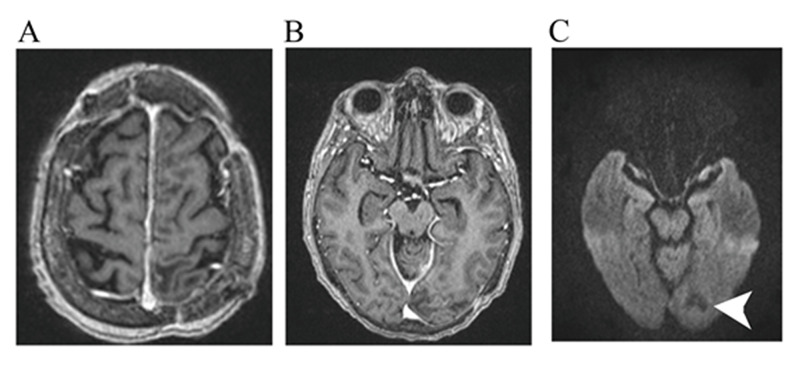
Three-month follow-up MRI. Three-month follow-up MRI of the brain with contrast (A-B) and DWI sequence (C) illustrating no abnormal enhancement of DWI signal (arrow) in the area corresponding the to prior subdural empyema and the abscess indicating subdural empyema, and the occipital abscess are completely resolved. MRI: magnetic resonance imaging; DWI: diffusion-weighted imaging

The patient was taken to the operating room for stereotactic drainage of the left subdural abscess, hydrogen peroxide irrigation, and drain placement in the left occipital abscess. Surgical culture showed *S. intermedius* once again. Post-operatively, 10 mg of vancomycin (2 mL of 5 g/mL solution) once a day was administered directly into the left occipital abscess through the drain daily for three days, along with 2 g of intravenous ceftriaxone every 12 hours for six weeks. At the three-month follow-up, MRI demonstrated a complete resolution of the abscess and subdural empyema without obvious neurological deficit (Figure 4).

## Discussion

Although intracranial abscesses are rare (0.9 per 100,000) [4], they continue to be a life-threatening condition. Early recognition is paramount to proper treatment and prevention of neurological compromise. However, presenting signs and symptoms of abscess are often nonspecific and vary greatly depending on the size and location of the lesion. One of the most common symptoms reported with brain abscesses is headaches, followed by altered awareness, fever, seizure, and focal neurological deficits [5]. The origin of some brain abscesses can be traced to previous maxillofacial infections, including sinusitis and mastoiditis or prior craniotomy; however, the etiology of most infections remains uncertain [6].

The majority of these abscesses result from infection due to gram-positive bacteria, most often *Staphylococcus aureus* and *S. milleri* [7]. Current empiric treatment of intracranial abscesses involves surgical resection of the lesion followed by intravenous broad-spectrum antibiotics, covering for gram-positive, gram-negative, and anaerobic bacteria [8]. However, there has been a progressive increase in the number of drug-resistant bacteria infecting brain parenchyma, resulting in higher rates of recurrence following initial intervention. These resistant organisms often require vancomycin and linezolid for adequate treatment [9].

*S. intermedius* is a common co-infector of intracranial abscesses, but rarely is it the predominant species [7]. When *S. intermedius* has been found to be the predominant bacteria in a brain abscess, it is usually secondary to hematogenous spread from a prior focus of infection (e.g., sinus, teeth, middle ear), most often seen in children [10]. However, the origin of *S. intermedius* intracranial abscesses in adults has historically been difficult to identify, as is the case with our patient [11]. In addition to the rarity of the predominance of this species in brain abscesses, studies have shown that *S. intermedius* has developed resistance to many commonplace antibiotics, including penicillin, cephalosporins, macrolides, ciprofloxacin, and clindamycin. Fortunately, there are antibiotics that have been shown to be consistently effective against *S. intermedius* such as vancomycin, teicoplanin, and imipenem [12,13].

Similar to other studies, our patient did not have a clear origin for her *S. intermedius* infection, with no history of recent infection that could have contributed to the formation of her intracranial abscess. Our patient did not respond entirely to her initial treatment with intravenous ceftriaxone and had a recurrence of her abscess. A possibility for the recurrence of the infection is due to inadequate levels of the drug at the target intracranial site. Studies have shown that ceftriaxone has decreased penetrability through the blood-brain barrier when there is a lack of meningeal inflammation, making it difficult to achieve adequate levels of the antibiotic in the brain parenchyma [14]. As the patient did not respond to standard intravenous antibiotic treatment and her health was deteriorating after primary intervention, we decided to treat her recurrence with vancomycin via an intracranial drain while continuing the intravenous treatment. The use of an intracranial drain as a conduit for drug administration has been shown to be a safe and viable option in other studies in the past [15]. We chose to use vancomycin rather than ceftriaxone as intrathecal administration of vancomycin has been well studied while the outcomes of intrathecal administration of ceftriaxone are not well known [16]. Furthermore, our initial study showed that the organism was susceptible to vancomycin, making this an appropriate choice. The combination of systemic and targeted therapy proved to be effective in our case as our patient showed resolution of her symptoms without recurrence.

## Conclusions

Brain abscesses can be challenging to diagnose early and have a complicated treatment regimen. Most cases are due to *S. aureus* and *S. milleri*, but here we presented a rare case of intracranial abscess caused by *S. intermedius*. Furthermore, due to the failure of traditional intravenous ceftriaxone and recurrence of the abscess, we used an uncommon technique of targeting the lesion site by administering vancomycin through a surgical drain. The combination of systemic and direct targeting of the intracranial abscess proved to be effective in the treatment of our patient’s recurrent infection.
